# Is It Rational to Study Coagulations Test Routinely before Operations and Invasive Procedure: Single Center Retrospective Study

**Published:** 2019-10-01

**Authors:** Hassan Mansouritorghabeh

**Affiliations:** Allergy Research Centre, Ghaem Hospital, School of Medicine, Mashhad University of Medical Sciences, Mashhad, Iran

 Dear Editor,

 I read with interest the recent paper by *Fergün Yılmaz and et al.* entitled: “ Is It Rational to Study Coagulations Test Routinely before Operations and Invasive Procedure: Single Center Retrospective Study” that has been published in the recent issue of International Journal of Hematology-Oncology and Stem Cell Research^   1 ^.

The function of the hemostasis system to prevent bleeding episodes is the result of collaboration and crosstalk between blood vessels, blood cells, coagulation factors and fibrinolysis system. Hence, the detection of aberrations involved in this complex system of hemostasis is cumbersome, complicated and expensive^   2 ^. Indeed, at present time we do not know a single test who can check all these four systems altogether. 

Recently, we retrospectively reviewed causes of death among patients with hemophilia in our region^   3 ^. In that survey, there were cases who had died or encounter serious problems after circumcision or other surgeries. Since there is no complete screening test that can be substituted for PT and APTT to detect hemorrhagic tendency, it seems that they are only available usable methods for detection of bleeding disorder, especially in developing countries, where there are restricted diagnostic facilities. Anyway, these tests would not be ordered as screening test to predict bleeding tendency^   4 ^. The necessity of performance of screening tests of hemostasis system before surgical procedures has remained a challengeable issue among hematologists and surgeons yet. 

Although family and personal history are the first steps of selection coagulation tests, it should be kept in mind that 1/3 of cases with hemophilia are de novo and without any familiar history of bleeding history in family^5^. Moreover, cases with a diagnosis of acquired hemophilia and other bleeding disorders have not only negative family history but also a negative personal history of bleeding before their recent hemorrhagic episode that terminated to the diagnosis of acquired bleeding^6^. 

The authors have concluded in abstract that they did not find any statistically significant difference regarding bleeding among two groups with high PT and PTT and low PT and PTT. This comparison has a significant bias as a number of patients (n=38, 67.8%) cancelled their surgeries and some others were not operated. 

The new version of hemostasis assays including, Thrombin generation test, Thromboelastography, etc. is better indicator of any abnormalities in the hemostasis cascade ^   2 ^. It takes time till the current diagnostic facilities to be available in developing countries, where there are a noticeable number of patients with bleeding disorders. At the present time, it seems that it would be rational to carry out the current screening tests of hemostasis, especially in the two groups: 1) Adults with no clear history of hemorrhagic events regarding as a challenge for the hemostasis system (minor or major surgery) in themselves or in first-degree members of their families; 2) All children without a history of any previous invasive procedure that may be associated with bleeding risk.
